# A Novel Method for Tracking Individuals of Fruit Fly Swarms Flying in a Laboratory Flight Arena

**DOI:** 10.1371/journal.pone.0129657

**Published:** 2015-06-17

**Authors:** Xi En Cheng, Zhi-Ming Qian, Shuo Hong Wang, Nan Jiang, Aike Guo, Yan Qiu Chen

**Affiliations:** 1 School of Computer Science, Shanghai Key Laboratory of Intelligent Information Processing, Fudan University, Shanghai, China; 2 State Key Laboratory of Neuroscience, Shanghai Institute of Biological Sciences, Chinese Academy of Sciences, Shanghai, China; 3 Jingdezhen Ceramic Institute, Jingdezhen, China; Alexander Fleming Biomedical Sciences Research Center, GREECE

## Abstract

The growing interest in studying social behaviours of swarming fruit flies, *Drosophila melanogaster*, has heightened the need for developing tools that provide quantitative motion data. To achieve such a goal, multi-camera three-dimensional tracking technology is the key experimental gateway. We have developed a novel tracking system for tracking hundreds of fruit flies flying in a confined cubic flight arena. In addition to the proposed tracking algorithm, this work offers additional contributions in three aspects: body detection, orientation estimation, and data validation. To demonstrate the opportunities that the proposed system offers for generating high-throughput quantitative motion data, we conducted experiments on five experimental configurations. We also performed quantitative analysis on the kinematics and the spatial structure and the motion patterns of fruit fly swarms. We found that there exists an asymptotic distance between fruit flies in swarms as the population density increases. Further, we discovered the evidence for repulsive response when the distance between fruit flies approached the asymptotic distance. Overall, the proposed tracking system presents a powerful method for studying flight behaviours of fruit flies in a three-dimensional environment.

## Introduction

Social behaviours of swarming fruit flies, *Drosophila melanogaster*, have recently attracted increasing scientific attention [1]. Such studies may deepen our understanding of the neural mechanisms underlying social behaviours [2]. The rising of such research activities is fueled by recent advances of the video tracking technology. The video tracking method can automatically measure individual’s motion states using videos from different camera views [3]. Previous studies have made contributions on developing methods for tracking fruit flies walking in planar arenas (2D) [4–9] or for tracking fruit flies flying in cubic or cylindrical arenas (3D) [10–14]. Collective behaviour [15], however, usually happens in a large group of animals, such as bird flocks [16, 17], fish shoals [18–20], and insect swarms [21–25]. Acquiring individual’s 3D motion state through time is still an open problem while the population size is large or the population density is high.

In order to obtain 3D quantitative motion data of swarming fruit flies, multiple synchronized and calibrated cameras are employed to capture videos. It needs methods to establish cross-view and cross-frame correspondence for methods to achieve the tracking purpose. Due to severe occlusion and mutually similar appearance, finding correspondences across multiple views is a severe challenge. And moreover, since we have to film the entire swarm in the cameras’ field-of-view (FOV), each fruit fly only takes up a few pixels in the images. Such images makes resolving pixels of body and wings more challenging. From this viewpoint, previous methods [14, 23, 24, 26, 27] may have the following limitations: (I) The detected result is unstable. Due to the fast wing strokes of fruit flies (less than 4 ms per wing stroke [28]), detected objects which include pixels of the wings and body decrease the accuracy on computing their barycenters; (II) The fruit fly’s orientation cannot be reconstructed. Since the pixels of body and wings cannot be resolved, there are no sufficient visual cues for estimating a fly’s orientation in 3D.

We propose in this paper a multi-camera tracking system for acquiring motion data (the location and orientation) of individuals of swarms of fruit flies through time. Videos of fruit flies were captured by three synchronized and calibrated high-speed cameras, in which fruit flies were flying in a confined cubic flight arena. We propose an approach to detect pixels corresponding to a fruit fly’s body and an algorithm for computing the fly’s orientation. The orientation information not only helps increasing the efficiency of tracking fruit flies but also helps validating and correcting the estimated motion state. The tracking system has successfully acquired individual motion data of hundreds of fruit flies through time. We show the results of experiments on five experimental configurations to demonstrate the opportunities that our system offers for generating high-throughput quantitative motion data. We performed quantitative analysis on the kinematics and the spatial structure and the motion patterns of fruit fly swarms. We found that there exists an asymptotic distance between fruit flies in swarms as the population density increases, and we discovered that fruit flies have a strong tendency to turn away when the distance between them approached the asymptotic distance. Overall, the proposed tracking system presents a powerful method for studying flight behaviours of fruit flies in a 3D environment.

## Methods

**Fig 1:**
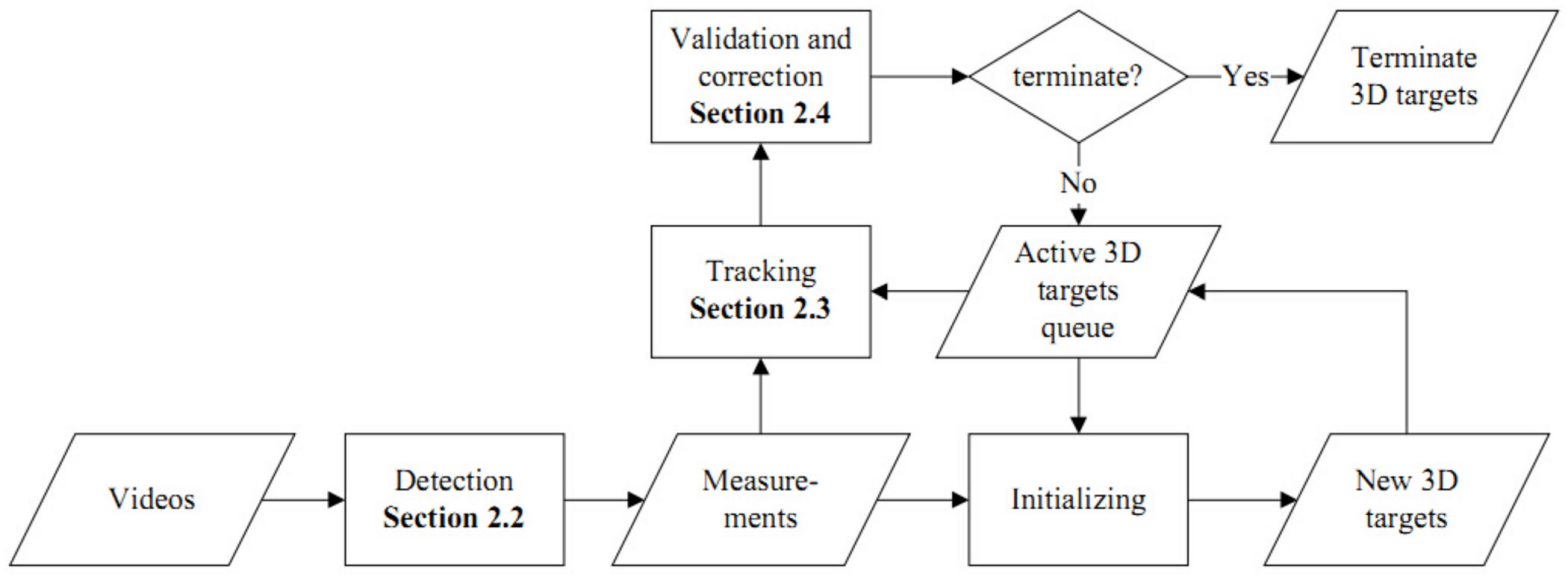
Diagram of the tracking system. Three steps are detailed in section 2 (Methods).

We have developed an experimental imaging system consisting of a transparent cubic flight arena, two planar lights, and three high-speed cameras (Section 2.1). The three cameras are employed to capture videos of flying fruit flies. Videos are the raw observation for the tracking system. Fig 1 shows the overview of the proposed tracking method. The first step of the system is to extract high level observations (*measurement*s) from the raw observation (Section 2.2). Measurements are input to the proposed tracking algorithm. At the tracking step (Section 2.3), the system exploits an “one-to-one” strategy, meaning that it creates (or invokes) one tracker to track one target using measurements associated with the target. After the motion state of a target has been estimated, the system invokes a validating and correcting process (Section 2.4) to achieve results that are more accurate.

### 2.1 Experimental imaging system

The flight arena is a Lucite cube of side length 360 mm (inner). The arena was built by gluing by five transparent acrylic planks with chloroform. A feeding-tube matched open-top sunroof in 50 mm diameter circle was handled on escape-proof inner besieged flange. Three monochrome high-speed digital video cameras (IO Industries Canada, Flare 4M 180-CL, 2040*v* × 2048*h* pixels at 100 fps) were placed approximately 900 mm from the cubic arena against two orthogonally placed back-lit cool-running lamps. Each lamp was made by LED arrays and covered by a diffusion sheet to generate gentle and flicker-free planar illumination. The cameras were mounted with 17–35 mm lens. The supporting information (S1 Fig) shows the experimental equipment.

### 2.2 Detection

**Fig 2:**
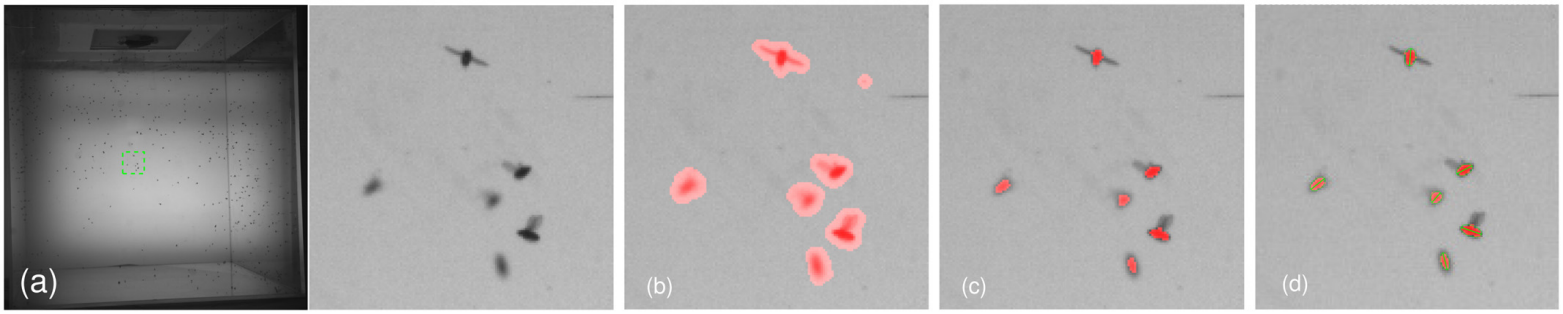
Detecting. (a) The image at the left was filmed by a back-lit camera and the patch marked by the green rectangle was zoomed at the right. (b) The patch was overlaid with red blobs which were detected using background subtraction. (c) The patch was overlaid with refined blobs in which the pixels of wings were removed. (d) The result of fitting each blob with an ellipse.

Previous studies [10–14, 23] have demonstrated that the illumination was usually provided by front-lighting in laboratory. The front-lighting means lights and cameras are placed at the same side of the targets. It has the advantage of showing the rich texture of targets and the background. The rich texture however makes target detection and resolving body’s pixels very difficult. The back-lighting is the opposite, *i.e*. the targets are imaged as silhouettes against plain white background. In our problem, we moved the lights to the side of targets opposite the cameras, meaning that targets were back-lit in camera views. Fig 2a shows an image in which fruit flies were back-lit in the camera view. We presented a simple and efficient detecting approach in this paper. The proposed approach makes full use of the different levels of transparency between the wings and body of a fruit fly, in which the pixels of a fly’s wings have higher intensities than pixels of its body. The proposed detecting approach segments an image into blobs. Each blob is an image area with pixels’ locations and intensities, and each blob represents the observation of a certain fruit fly in a camera view. To simplify notation, all definitions in this section ignore subscripts for cameras and moments.

#### 2.2.1 Segmenting blobs

In the first step, we subtract a Gaussian background model from an acquired image. Given a pixel *i* which denotes the location of a certain pixel in a certain camera view, let *μ*(*i*) be its mean intensity and *σ*(*i*) be its standard deviation of intensities through time. We compute *μ*(*i*) and *σ*(*i*) according to a sequence of consecutively acquired images in the camera view. That is, *μ* and *σ* define the Gaussian background model in the camera view.

The pixel *i* in a certain acquired image is segmented as a foreground pixel if its intensity satisfies the constraint:

$$\begin{array}{c} \frac{I(i)-\mu (i)}{\sigma (i)}<-C_{1} \end{array}$$ (1)

where *I* denotes the acquired image. Connected foreground pixels are clustered as blobs *b* ∈ {1, …, *b*_*n*_}. We choose the constant value *C*_1_ = 1.5 which guarantees that each blob includes pixels of a fly’s wings and body. Fig 2b shows the segmented blobs of the image patch.

#### 2.2.2 Removing wings pixels

Fruit flies can make wing-strokes very fast (*e.g*. less than 4 ms [28] for one wing-stroke), meaning the wings’ positions between consecutive frames are inconsistent unless the camera’s frame-rate is very high, such as 2,000 frames per second. That is unusual for conventional multi-camera system. From this viewpoint, methods, such as [14, 27], which included the wings pixels in the detecting results were not precise enough.

In the second step, we use local Gaussian models to remove pixels of wings from blobs. Given a blob *b*, let *μ*_*b*_ denote its mean intensity and *σ*_*b*_ denote its standard deviation of the intensities. These variables, *μ*_*b*_ and *σ*_*b*_, are computed using all pixels belonging to the blob *b*. The local Gaussian model of the blob *b* is thereby defined by *μ*_*b*_ and *σ*_*b*_. This second step is a refinement step and is computed as

$$\begin{array}{c} blob\left(b,i\right)=\{\begin{array}{cc} 1 & \frac{I\left(i\right)-\mu_{b}}{\sigma_{b}}<-C_{2}\\ 0 & otherwise \end{array} \end{array}$$ (2)

where *b* ∈ {1, …, *b*_*n*_} denotes the blobs and *i* ∈ {*i*_*b*,1_, …, *i*_*b*,*n*_} denotes the pixels of a certain blob *b*. If *blob*(*b*, *i*) is equal to 0, we remove the pixel *i* from the blob *b*. We choose the constant value *C*_2_ = 1.5 which guarantees most of pixels of a fly’s body being preserved after refinement. Fig 2c shows the image patch overlaid with the refined blobs. This step makes full use of the different levels of transparency between a fruit fly’s wings and its body (*i.e*. the pixels of a fly’s wings have higher intensities than those of its body). This is achieved thanks to the fruit flies were back-lit in camera views.

#### 2.2.3 Fitting blob with ellipse

In order to fit a blob with an ellipse accurately, it is usually that fitting a 2D Gaussian to the locations of all pixels in the blob. Given the parameters of the best-fitting Gaussian, the parameters of the ellipse can be computed. Instead of just computing the mean and covariance of all pixels in the blob, we compute a weighted mean and covariance. Let *w*(*i*) denote the weight of pixel *i*, the weight *w*(*i*) is defined as the normalized distance of the pixel *i*’s intensity to the background model which is defined by *μ* and *σ*:

$$\begin{array}{c} w\left(i\right)=\frac{\left|I(i)-\mu (i)\right|}{\sigma (i)} \end{array}$$ (3)

Therefore, the weighted mean and covariance of the pixels of the blob *b* are computed as:

$$\begin{array}{ccc} \mu_{b}^{\prime } & = & \frac{1}{W}\sum_{i}w\left(i\right)*i,\;i\in \left\{i_{b,1},...,i_{b,n}\right\} \end{array}$$ (4)

$$\begin{array}{ccc} \Sigma_{b}^{\prime } & = & \frac{1}{W}\sum_{i}w\left(i\right)*\left(i-\mu_{b}^{\prime }\right){\left(i-\mu_{b}^{\prime }\right)}^{T},\;i\in \left\{i_{b,1},...,i_{b,n}\right\} \end{array}$$ (5)

where *W* = ∑_*i*_ *w*(*i*), *i* ∈ {*i*_*b*,1_, …, *i*_*b*, *n*_} defines the weight’s normalization constant, and *i* ∈ {*i*_*b*,1_, …, *i*_*b*,*n*_} denotes the pixels belonging to the blob *b*. The weighted mean and covariance not only improve the robustness to non uniform illumination (*e.g*. a single threshold for all images), but also give us the sub-pixel accuracy on fitting ellipses. The mean $\mu_{b}^{\prime }$ defines the center of the ellipse, and the covariance $\Sigma_{b}^{\prime }$ defines the axis and the direction of the ellipse. Fig 2d shows the results of fitting each blob with an ellipse.

#### 2.2.4 Measurements definition

We refer to a blob and the ellipse fitted to it as a ***measurement***. Let *χ* denote a ***measurement***, each measurement includes two components:

$$\begin{array}{c} \chi =\{b,e(b)\} \end{array}$$ (6)

where *b* denotes the blob with pixel locations and intensities, and *e*(*b*) defines the ellipse fitting to the blob. We used 3 cameras in our experiments and therefore the set of all available measurements at a certain moment was defined as {*χ*_*v*,*i*_ | *v* ∈ {1, 2, 3}, *i* ∈ {1, …, *i*_*v*,*n*_}}.

### 2.3 Tracking

**Fig 3:**
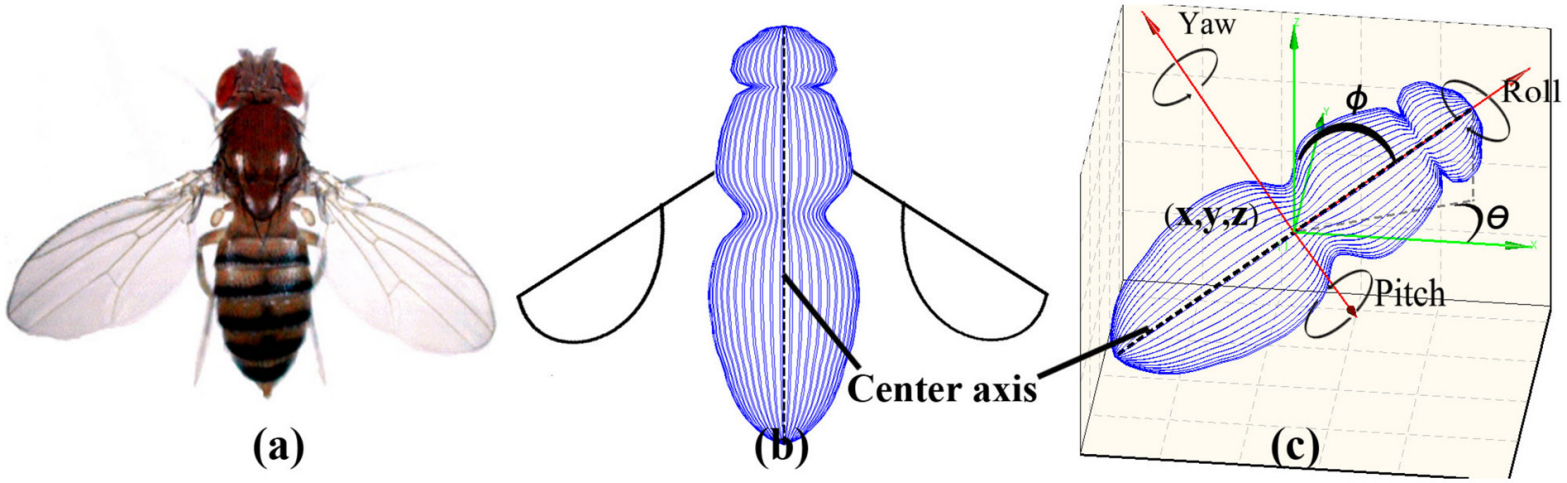
The fruit fly’s model. (a) The front image of an adult fruit fly. (b) The articulated model of the fruit fly. (c) A fruit fly locates at (*x*, *y*, *z*). Its orientation is defined by two angles (*θ*, *ϕ*) against the world’s coordinate system. *θ* is an angle from x-axis and *ϕ* is an angle from horizontal (the x-y plane). Combining the location (*x*, *y*, *z*) and orientation (*θ*, *ϕ*), it defines a directed line-segment in 3D space, the *center-axis* of the fruit fly.

Previous studies [10–14, 23] have proposed methods that model targets as points and estimating only targets’ locations in 3D. In order to encompass orientation information, we model a target as a directed line-segment in 3D (the *center-axis* of a target). Fig 3 shows the modeling approach. The fruit fly in our system is denoted by $\left\{x,y,z,\theta ,\phi ,\tilde{l}\right\}$ where $\tilde{l}$ is a constant scalar. The constant scalar $\tilde{l}$ denotes the length of *center axis* (In our problem, $\tilde{l}=2.73$ mm is the average body length of fruit flies). Here (*x*, *y*, *z*) denotes the location of the fruit fly and (*θ*, *ϕ*) denotes its orientation.

**Fig 4:**
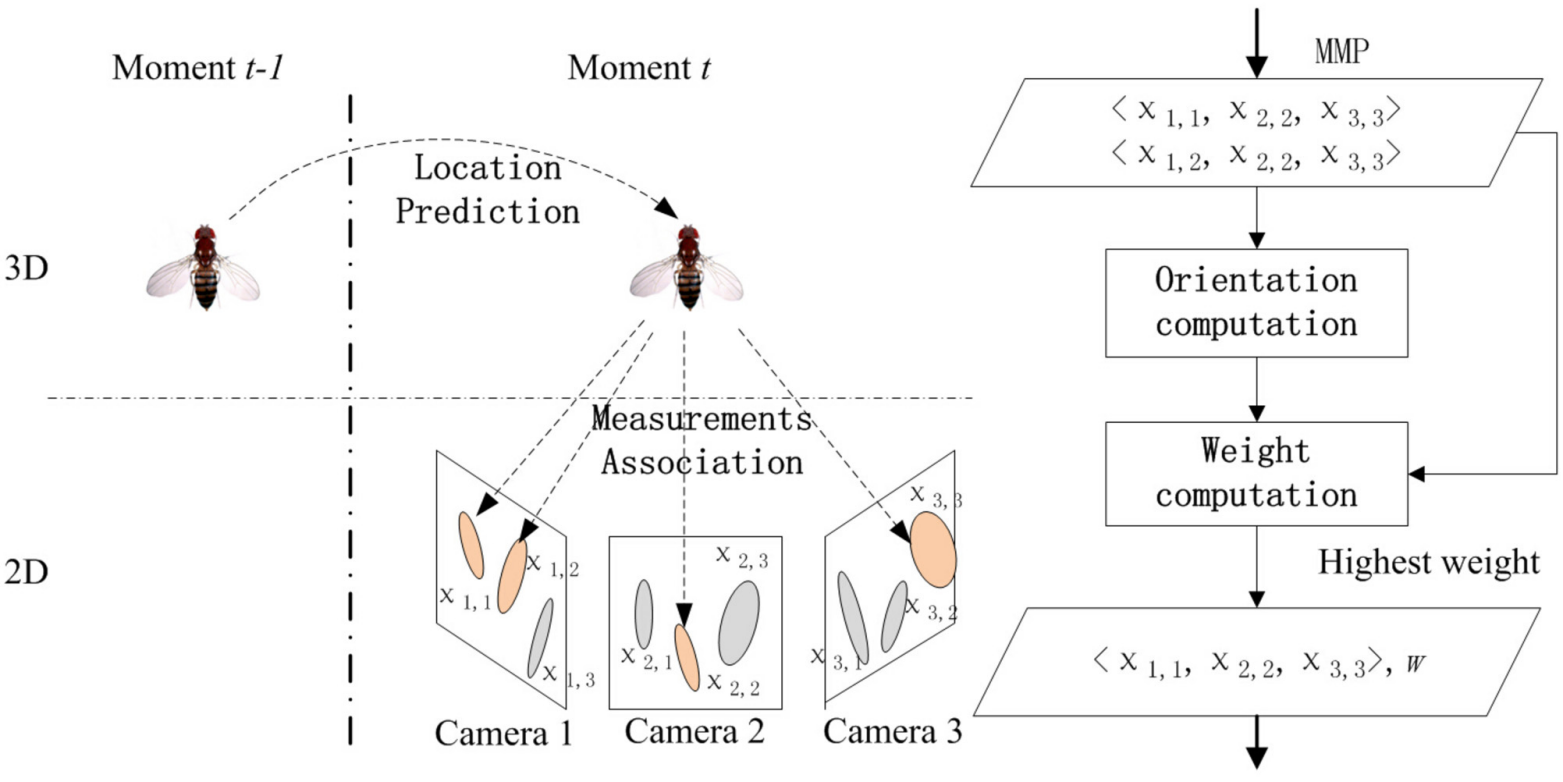
Overview of the tracking algorithm. By exploiting the particle filtering solution, we use ${\tilde{n}}^{P}=200$ particles sampling the posterior distribution of the motion state of a fruit fly at moment *t*. Each particle includes two components: the fly’s location (*x*, *y*, *z*) and the fly’s orientation (*θ*, *ϕ*). The location component is predicted by using the dynamic model and the fly’s location at moment *t*−1. After the location component is predicted, an MMP is associated with the particle. Then the orientation component is computed using the MMP. And then the tracking algorithm computes the weight of the particle. If there are more than one MMP (*e.g*. since the target is associated with two measurements in *Camera 1*, the algorithm thereby generates two MMPs (*χ*_1,1_, *χ*_2,2_, *χ*_3,3_) and (*χ*_1,2_, *χ*_2,2_, *χ*_3,3_)), the algorithm choose the MMP having highest weight (*e.g*. (*χ*_1,1_, *χ*_2,2_, *χ*_3,3_)) and the orientation computed by the MMP. Finally, the motion state of the fly at moment *t* is the expectation computing by the weighted particles.

The proposed tracking algorithm is based on the Bayesian inference framework. Fig 4 shows the overview of the tracking algorithm. In our tracking algorithm, the motion state of targets propagates (location (*x*, *y*, *z*) is predicted and orientation (*θ*, *ϕ*) is computed explicitly) in 3D space through time. The location of a target (*x*, *y*, *z*) at moment *t* is predicted by the dynamic model conditioned on its location at moment *t*−1. We developed a measurements association algorithm which associates 2D measurements in all views with a 3D target after its location being predicted at each moment. Measurements which have been associated with a certain target generate a **m**atched **m**easurement **p**air (MMP). The orientation of the target (*θ*, *ϕ*) is then computed using the MMP. The predicted location (*x*, *y*, *z*) and computed orientation (*θ*, *ϕ*) form a sampled motion state of the target (a particle). The probability of each particle is evaluated by incorporating in the MMP of the target at moment *t*. In brief, the proposed tracking algorithm is a particle filtering solution for a Bayesian inference problem.

#### 2.3.1 Location prediction

We adopt the Bayesian tracking framework and construct the following eight-dimensional model to define the system state

$$\begin{array}{c} \left\{x,y,z,x^{-},y^{-},z^{-},\theta ,\phi \right\} \end{array}$$ (7)

where the “^−^” superscripted terms denote the location of the target at previous moment.

In order to reduce the state space, we partition the state space into two parts: the location state *X*(*x*, *y*, *z*, *x* ^−^, *y* ^−^, *z* ^−^) and the orientation state *O*(*θ*, *ϕ*). We thereby predict a target’s location state using the dynamic model and the target’s location state at moment *t*−1, and leave the orientation state being computed explicitly.

We adopt the first-order linear extrapolation (FLE) as the dynamic model. The FLE model assumes that the next location of a target is defined by the linear extrapolation of the last two locations. The transition function is defined as:

$$\begin{array}{c} X_{t}=[\begin{array}{c} 2I_{3}\;\;\;-I_{3}\\ I_{3}\;\;\;\;\;\;0_{3} \end{array}]X_{t-1}+v_{t} \end{array}$$ (8)

where *I*_3_ is a 3 × 3 identity matrix and *v*_*t*_ ∼ 𝒩(0, Σ) is the transition noise.

Let *Z*_1:*t*_ = {*Z*_*i*_, *i* = 1, …, *t*} denote the set of all available observation up to moment *t*, the Bayesian inference can be formulated as a problem of estimating the posterior probability *p*(*X*_*t*_|*Z*_1:*t*_) [29]. Under the first-order Markov assumption and the Bayes’ rule, we can get the well-known equation of Bayesian filtering

$$\begin{array}{c} p\left(X_{t}|Z_{1:t}\right)\propto p\left(Z_{t}|X_{t}\right)\int p\left(X_{t}|X_{t-1}\right)p\left(X_{t-1}|Z_{1:t-1}\right)dx_{t-1}, \end{array}$$ (9)

where *p*(*Z*_*t*_|*X*_*t*_) is called the observation model. In our problem, $Z_{t}={\left\{Z_{t}^{v}\right\}}_{v=1}^{3}$ denotes the collection of measurements from all 3 camera views at moment *t*.

By using the particle filtering solution for the Bayesian inference problem, the posterior of a target’s motion state at moment *t*, *p*(*X*_*t*_|*Z*_1:*t*_), is approximated by a set of weighted particles: $\left\{(s_{t}^{n},w_{t}^{n})|n\in 1...{\tilde{n}}^{p}\right\}$. We set ${\tilde{n}}^{p}=200$ in our experiments. Each particle $s_{t}^{n}$ is associated with a weight $w_{t}^{n}$ which is proportional to the likelihood of the sampled motion state given the observation at moment *t*, in which the sampled motion state is represented by the particle. New particles are drawn from particles in the previous step using importance sampling [29] and move independently according to the dynamic model. Given a set of particles $\left\{(s_{t}^{n},w_{t}^{n})|n\in 1...{\tilde{n}}^{p}\right\}$, the estimated motion state of the target at moment *t*, ${\hat{X}}_{t}$, is computed as the expectation

$$\begin{array}{c} {\hat{X}}_{t}=E\left(X_{t}|Z_{1:t}\right)=\sum_{n=1}^{{\tilde{n}}^{p}}w_{t}^{n}s_{t}^{n} \end{array}$$ (10)

In our problem, each particle is defined as $s_{t}^{n}=(X_{t}^{n},O_{t}^{n},\tilde{l})$. At this point, we have the incomplete particle $s_{t}^{n}=(X_{t}^{n},*,\tilde{l})$. The * denotes the uninitialized value.

#### 2.3.2 Measurement association

At each moment, many measurements are extracted from images. We have to decide which measurement is the observation of a certain target in a certain camera view. That is, a measurement has to be assigned to a target if the target is “observed” in that camera view. This is the task of measurement association, and we propose the **p**ixels **o**ccupancy **t**est (POT) algorithm for solving the task. The POT algorithm simultaneously solves the association problem across all camera views. The POT algorithm is based on the idea of probability gating. A gate is a surface with constant probability [30, 31]. In our work it should be an ellipsoid according to the shape of a fruit fly. However, here we employ a sphere gate since there is no orientation state at this point. Let Γ define the association between the particle $s_{t}^{n}(X_{t}^{n},*,\tilde{l})$ and measurements *χ*_*v*,*i*_, *v* = 1..3, *i* = 1..*i*_*v*,*n*_. It is computed as

$$\begin{array}{c} \begin{array}{c} \Gamma \left(X_{t}^{n}\right)=\left\{\left(\chi_{v,i},\eta \right)|\eta >\eta_{c},v=1..3,i=1..i_{v,n}\right\}\\ \eta =\frac{card(\Omega \left(X_{t}^{n},\chi_{v,i}\right))}{card(\chi_{v,i}\left(b\right))}\;\;\;\;\;\;\;\;\;\;\;\;\;\;\;\;\;\;\;\;\;\;\;\\ \Omega \left(X_{t}^{n},\chi_{v,i}\right)=\left\{P^{v}{ϒ}^{3}\right\}⋂\chi_{v,i}\left(b\right)\;\;\;\;\;\;\;\;\;\;\;\;\;\;\;\;\;\;\;\;\;\;\;\;\;\;\;\;\;\;\;\;\; \end{array} \end{array}$$ (11)

where *card*(⋅) defines the function which counts the number of elements in a set of pixels, and *χ*_*v*,*i*_(*b*) denotes the blob’s pixels of the measurement *χ*_*v*,*i*_. Here ϒ^3^ denotes the discrete 3D points sampled from the surface of a sphere which locates at $X_{t}^{n}(x,y,z)$ with the diameter equal to $\tilde{l}$; and **P** ^*v*^ is the projection matrix of camera *v*. Therefore, $\Omega (X_{t}^{n},\chi_{v,i})$ defines the common pixels between *χ*_*v*,*i*_(*b*) and pixels which are projected into camera *v* from ϒ^3^; and *η* is proportional to the rate of common pixels, *η* ∈ [0, 1].

In Eq (11), *η*_*c*_ is a scalar positive-valued percolation threshold which varies from 0 to 1 for leveraging the restriction on association. Larger *η*_*c*_ means more restrictive association. In practice, we prefer *η*_*c*_ = 0.5 in the first tens of frames. And then *η*_*c*_ is set to a smaller value *η*_*c*_ = 0.25. Particles, which cannot associate with one measurement in any views, are labeled as “missing particle” and thereby ignored, *i.e*. the association had to succeed across all camera views. Besides, since the perspective effect causes many 2D occlusions in dense population it is acceptable that one measurement is associated with two or more particles.

#### 2.3.3 Orientation computation

**Fig 5:**
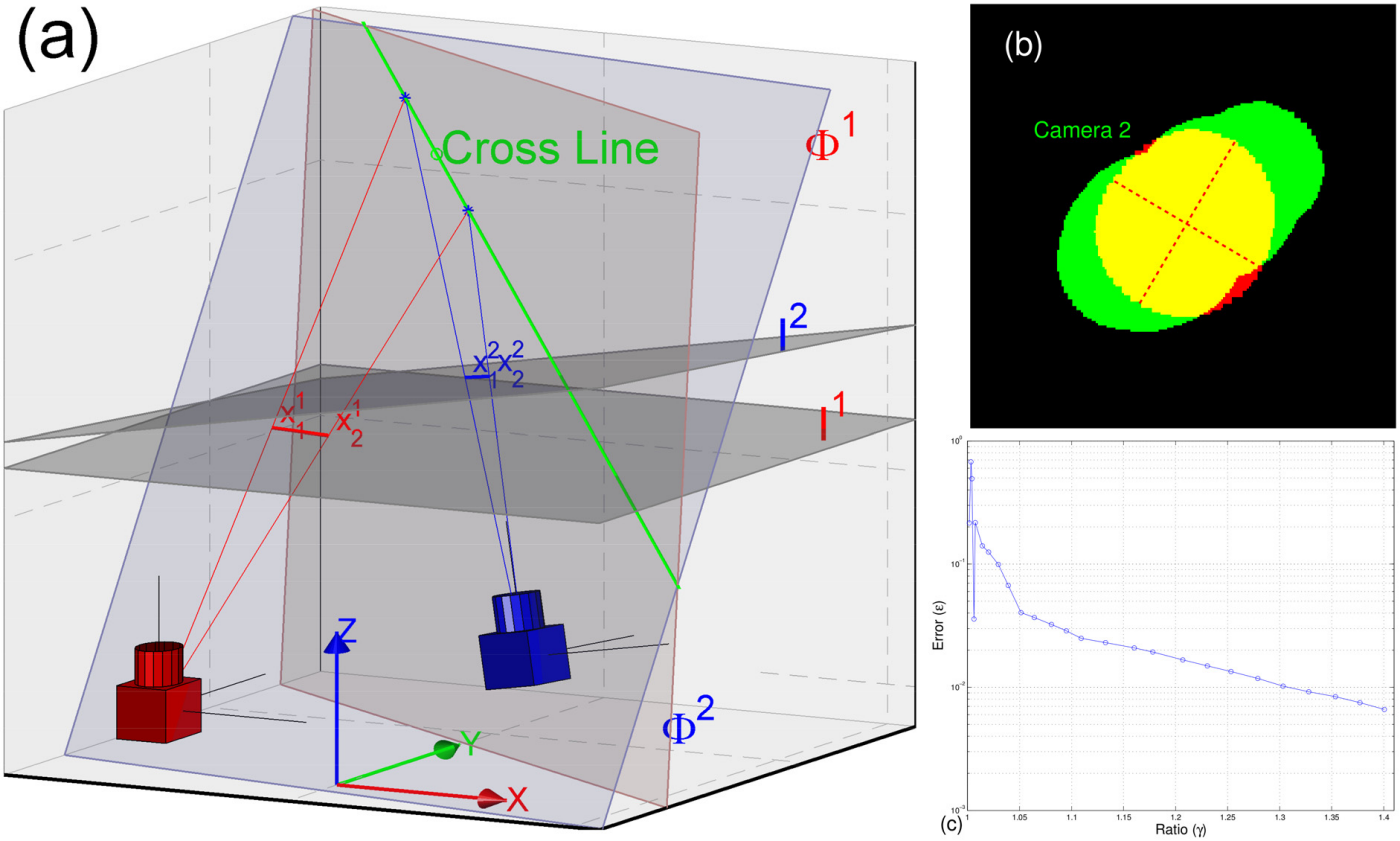
Orientation computation. (a) The geometric interpretation of the orientation computation. Here *I* ^1^ and *I* ^2^ denotes the image plane of *Camera 1*(red) and *Camera 2*(blue) respectively. The line-segment $\left\langle x_{1}^{1},x_{2}^{1}\right\rangle$(red) denotes the ellipse’s major axis in *I* ^1^. And the line-segment $\left\langle x_{1}^{2},x_{2}^{2}\right\rangle$(blue) denotes the ellipse’s major axis in *I* ^2^. The projection rays of those end-points define two plane Φ^1^ and Φ^2^ respectively, *e.g*. two red rays define Φ^1^. The orientation $O_{t}^{n}$ can be computed from the direction of the cross line (the longer green line-segment) between two planes Φ^1^ and Φ^2^. All definitions are defined in the world coordinate system. (b) The problematic orientation computation. The measurement is drawn in red, and red dashed lines are the major-axis and minor-axis of its ellipse. A generative shape according to the particle state $\left(X_{t}^{n},O_{t}^{n},\tilde{l}\right)$ is re-projected into *Camera 2* (see S2 Fig, the generative shape model). The green pixels are re-projected pixels and the yellow pixels are identical pixels. (c) The error *ϵ* as a function of the ratio *γ*. Here *γ* defines the length ration between the major-axis and the minor-axis of an ellipse.

Measurements defined by $\Gamma (X_{t}^{n})$ form an MMP (*c.f*. There may probably be more than one MMP. Please see Fig 4, the legend.). An MMP includes one measurement in each camera view. The ellipses of the MMP are employed to compute the target’s orientation according to the pinhole camera model in Euclid geometry. The geometric interpretation of computing the orientation is shown in Fig 5a.

Fig 5a shows the orientation $O_{t}^{n}$ of the particle can be computed from the direction of the cross line between two planes Φ^1^ and Φ^2^. However, the direction of the cross line may become problematic while the measurement is close to circular. If the measurements of an MMP are close to circular, the major axis of ellipse might have nothing to do with the plane in which the *center-axis* lies (as shown in Fig 5b). Let *γ* define the ratio between the major-axis to the minor-axis of an ellipse (*i.e*. *γ* measures the level of circle for a measurement), and let *α* define the angle between the computed orientation and the real one. We compute the error *ϵ* as *ϵ* = 1−*cos*(*α*). Fig 5c shows the error *ϵ* as a function of the ratio *γ*. It suggests the error *ϵ* is less than 0.01 while *γ* satisfies *γ* ≥ 1.3. In our case, we can choose two ellipses from an MMP (it includes 3 measurements from 3 camera views), which all satisfy *γ* ≥ 1.3. That is, the orientation $O_{t}^{n}$ can be computed accurately. At this point, we have the particle $s_{t}^{n}$ been completed: $s_{t}^{n}=(X_{t}^{n},O_{t}^{n},\tilde{l})$.

#### 2.3.4 Weight computation

The particle $s_{t}^{n}=(X_{t}^{n},O_{t}^{n},\tilde{l})$ represents a sampled motion state of a certain target at moment *t*. As aforementioned, after the location state $X_{t}^{n}$ having been predicted, the orientation state *O*_*t*_ is computed independently at moment *t*. That is, $w_{t}^{n}$, the weight of the particle can be computed as:

$$\begin{array}{c} w_{t}^{n}\propto p\left(Z_{t}|X_{t}^{n},O_{t}^{n}\right)=p_{ol}\left(Z_{t}|O_{t}^{n}\right)p_{al}\left(Z_{t}|X_{t}^{n}\right) \end{array}$$ (12)

where *Z*_*t*_ denotes the observation at moment *t* and is defined by $\Gamma (X_{t}^{n})$. The weight includes two factors: *p*_*ol*_, the orientation likelihood; and *p*_*al*_, the appearance likelihood.

**Orientation Likelihood** *p*_*ol*_ defines the likelihood which is computed using the orientation’s temporal consistency between consecutive moments. Let the target’s orientation at moment *t*−1 be *O*_*t*−1_(*θ*, *ϕ*), this likelihood is computed as

$$\begin{array}{c} p_{ol}\left(Z_{t}|O_{t}^{n}\right)\propto \exp\left(-\frac{\vec{d}\left(O_{t}^{n}\left(\theta ,\phi \right)\right)\cdot \vec{d}\left(O_{t-1}\left(\theta ,\phi \right)\right)}{∥\vec{d}\left(O_{t}^{n}\left(\theta ,\phi \right)\right)∥∥\vec{d}\left(O_{t-1}\left(\theta ,\phi \right)\right)∥}\right)\\ \vec{d}\left(\theta ,\phi \right)={\left(\sin(\phi )*\cos(\theta ),\sin(\phi )*\sin(\theta ),\cos(\phi )\right)}^{T} \end{array}$$ (13)

where $\vec{d}\left(\cdot \right)$ defines the direction vector of a target’s orientation.

**Appearance likelihood** *p*_*al*_ defines the likelihood which is computed using the appearance’s temporal consistency between consecutive moments. The target’s appearance is the blobs of the MMP which is associated with the target. The intensities of all pixels in the blob jointly encode features of a target’s intrinsic appearance, depth, background and illumination. These factors are approximately constant for consecutive frames while the target were filmed by high-speed cameras. The likelihood is thereby computed as

$$\begin{array}{c} p_{al}\left(Z_{t}|X_{t}^{n}\right)=\prod_{v=1}^{3}\exp\left(\eta *ncc\left(\chi_{v,i},appearance\left(v\right)\right)\right),\;\left(\chi_{v,i},\eta \right)\in \Gamma \left(X_{t}^{n}\right) \end{array}$$ (14)

where *ncc*(⋅) is the function to compute normalized cross correlation coefficient of the blobs of two measurements. Here, function *appearance*(*v*) denotes the function to get the appearance of the target in camera view *v*. The target’s appearance is updated and stored at moment *t*−1.

### 2.4 Validation and correction

At the end of the tracking process, the motion state $\left(X_{t},O_{t},\tilde{l}\right)$ of a certain target is computed according to Eq (10) at moment *t*. In order to validate the motion state of a target automatically, we employ the POT algorithm (*c.f*. Section 2.3.2) again. The POT algorithm associates measurements with a certain target across camera views, but here the discrete 3D points ϒ^3^ in Eq (11) are now sampled from the surface of a generated shape. We implemented a parameterized generative shape ${ϒ}^{3}(X_{t},O_{t},\tilde{l})$ in term of a profile curve revolving around the *center-axis* (see S2 Fig, the profile *ρ* and the generative shape $ϒ(X_{t},O_{t},\tilde{l})$). The *center-axis* is a line-segment which is defined by $\left\{X_{t}(x,y,z),O_{t}(\theta ,\phi ),\tilde{l}\right\}$. A similar idea of generating shape model had been presented by Fontaine *et al*. [32].

In order to invoke restrictive validation, we choose *η*_*c*_ = 0.8 (*c.f*. Eq (11)). The validation is successful if the target has been associated with at least one measurement in each camera view; otherwise failures. If the target is associated with more than one measurement in a certain camera view, we choose the most likely measurement. The successful result forms only one MMP. In the second part of this step, we reconstruct a candidate 3D location by triangulation using the ellipse’s center of each measurement in the MMP. If the distance between the target 3D location and the candidate 3D location is larger than $\tilde{l}$, we update the 3D location of the target using the average of these two locations. The last part of this step is to update and to store the appearance of a target. The appearance of a target is the MMP with a time stamp. The tracking system updates and stores the appearance of an active target in run-time memory.

## Experiments and Results

The cameras were calibrated using the method proposed in [33, 34] and synchronized by a hardware timing mechanism. All videos were captured at 100 frames per second. The captured videos were first stored in DVR Express® and then exported as *jpeg* format images (IO Industries Canada, DVR Express® Core Camera Link Full, monochrome, 10 × 8 bit, Full, 1*TB* × 3). The capturing and exporting processes were controlled by the Core View™ online console software. All software was running on a computer with Intel® Core™ i5-2500 CPU 3.3GHz and 8GB RAM. The proposed tracking method was implemented in the MATLAB™ environment with “Machine Vision Toolbox” [35]. The evaluation of tracking performance is provided in S1 File and the raw code is provided in S2 File.

### 3.1 Experiment set up

#### 3.1.1 Animals

Wild-type Canton fruit flies were fed basic medium (corn flour broth with brown sugar) 3–5 days after eclosion. The flies were housed at 25°C room temperature and 60% relative humidity under 12 hour light-dark circadian clock without sexual discrimination. The number of flies in each feeding tube was estimated at 120–150 individuals at a normal hatchability ratio after a three-day oviposition by the fresh postnatal parental generation.

#### 3.1.2 Imaging

The fruit flies were housed in a transparent cubic flight arena, and videos were captured by three back-lit cameras. We have collected videos according to five experiment configurations. For each session, the fruit flies were totally refreshed. At the beginning of each session, we added tubes (*c.f*. The fruit flies are originally in the feeding tube.) of flies to the arena through the open-top sunroof (see S1 Fig). The course of flight usually lasted for tens of seconds. We filmed the entire course of flight on each session and discarded the first 2 seconds (200 frames) of each video to compensate for bias on flight behaviours. Table 1 shows the list of videos. The lengths of each video varied, but all were longer than 30 seconds (*i.e*. 3000 frames).

**Table 1:** Video sets and raw results.

video set id	# fruit flies	configuration	# trajectories		# NND	Demo
			≥ 1*sec*	≥ 3*sec*		
T01091009401	≈ 150	*T01*	994	246	63,306	S1 Video
T02091009601	≈ 300	*T02*	1447	435	96,225	S2 Video
T03091009701	≈ 400	*T03*	2518	618	184,339	S3 Video
T04100309901	≈ 500	*T04*	3578	791	247,509	S4 Video
T05100310101	≈ 600	*T05*	4693	916	318,875	S5 Video

### 3.2 The raw data

During experiments, we found that many fruit flies flew intermittently in the arena. The reason, we believe, is probably the absence of continuous visual stimuli or olfaction stimuli. Our system does not attempt to maintain the identity consistency on fruit flies over extended durations. In this study, if a fruit fly disappeared, for example it landed on a wall for a while, it was labeled with a different identity by the tracking system when the fly appeared again. As a result, the consecutive motion states of a fruit fly (with a unique identity) mainly continued for hundreds of frames, although we have captured rather longer video sequences (thousands of frames). The sequence of consecutive motion states are usually called trajectories. Table 1 shows the number of trajectories of each configuration. We prefer to call these results, which known as trajectories, the raw data (see S3 Fig, which shows the snapshot of the raw data of a typical configuration, *T03*).

### 3.3 Velocity statistics

**Fig 6:**
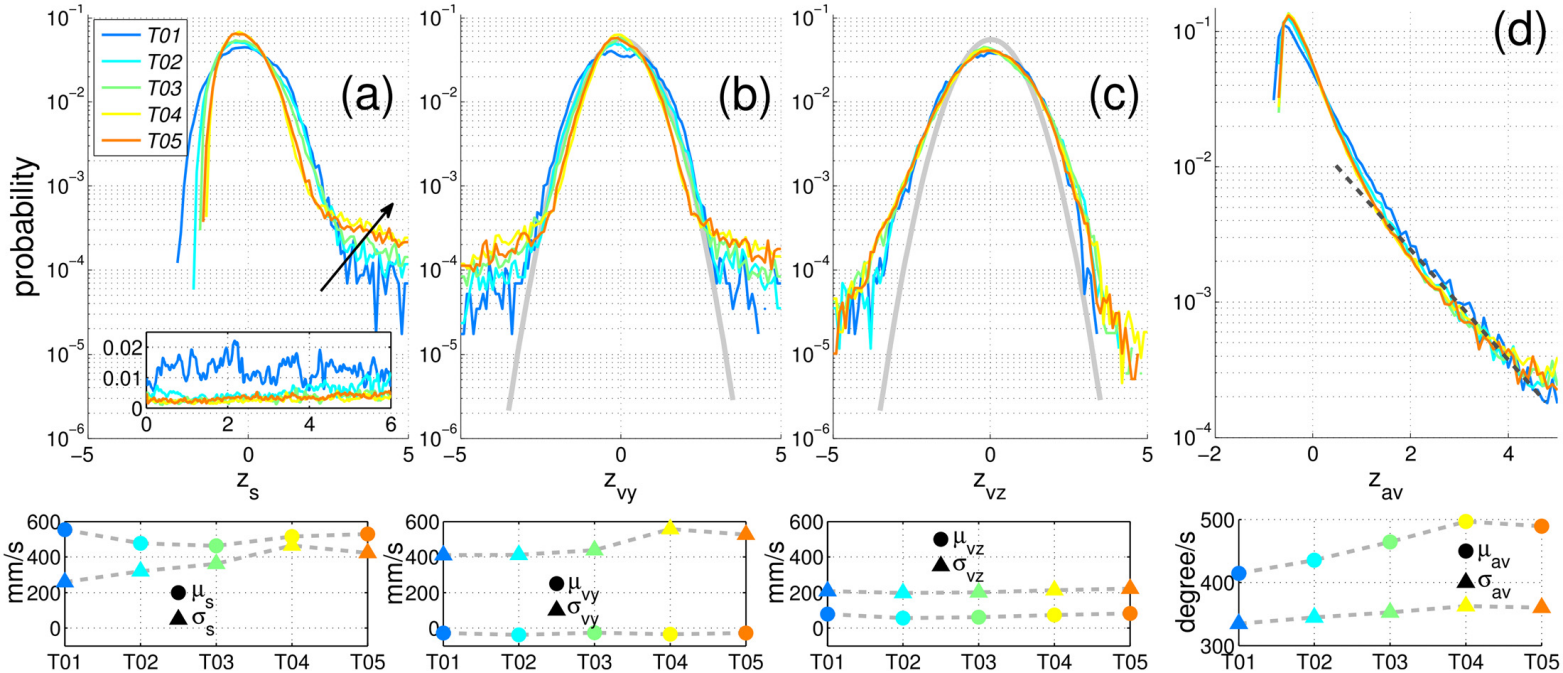
Statistics of fruit flies’ kinematics. The measured data of different configurations are color coded. The upper row shows the PDFs of measured z-scores *z* and the lower panel shows the measured mean *μ* and standard deviation *σ*. (a) The PDFs of z-scores of speed, *z*_*s*_, of all configurations. The black arrow shows the direction of growing population size. The inset shows the polarisation value in six seconds (more data is not present). (b, c) The PDFs of z-scores of velocity components: (b) the horizontal component, *z*_*vy*_, and (c) the vertical direction, *z*_*vz*_, of all configurations. The distributions are nearly Gaussian (an empirical Gaussian curve is shown in grey), with small deviation in the tails. (d) The PDFs of z-scores of angular velocity, *z*_*av*_, of all configurations. The dashed-line shows the nearly exponential long tails on the high angular velocity.

Since each fruit fly’s motion data has been obtained through time, we can thereby compute fruit flies’ kinetic measurement, such as velocity *v*, angular velocity *av* and speed *s* which is the magnitude of velocity *v* at each moment. Fig 6a shows the statistics of speed. It shows that the measured mean speed *μ*_*s*_ is greater than 400 mm/s (see the lower panel of Fig 6a), while the measured standard deviation *σ*_*s*_ increases from 200 mm/s to 400 mm/s as the population size increases. Considering the z-score (*a.k.a* the standard score) of speed denotes the fluctuation of speed, we computed the z-scores of all configurations and reported the PDFs of z-scores of each configuration in Fig 6a (the upper panel). The z-score of speed *z*_*s*_ is computed as *z*_*s*_ = (*s*−*μ*_*s*_)/*σ*_*s*_. Fig 6a shows the fluctuation of fruit flies’ speed usually narrows down (*i.e*. the amplitude of fluctuation decreases) as the population size increases. That is, the flight of fruit flies in a denser population environment is less volatile than those in a less dense environment. It suggests fruit flies have probably awareness of the environment. The black arrow in Fig 6a (the upper panel), which points to the direction of growing population size, shows the long tails of speed fluctuation. These tails are nearly exponential and grow monotonically with the population size, which suggest that fruit flies in a denser population environment perform faster manoeuvres. It probably are these long tails which cause the standard deviation of speed increases as the population size increases. On the other hand, previous studies [36, 37] have demonstrated that the fruit fly exhibits a flight pattern in which straight flight sequence interspersed with rapid turn called saccades. Fig 6d shows the statistic of angular velocity. The measured mean angular velocity *μ*_*av*_ is greater than 400 degree/s (see the lower panel of Fig 6d), while Fig 6d (the upper panel) shows that the angular velocity *av* is less than the mean *μ*_*av*_ in most of moments. That is, the angular velocity is usually less than 400° per seconds. But the nearly exponential tails (indicated by dashed-line in grey) suggest that fruit flies have also often taken the rapid turn.

Considering the velocity components, Fig 6b and 6c show the statistics of velocity components: *vy* (the horizontal velocity component, the other horizontal velocity component *vx* is statistically the same) and *vz* (the vertical velocity component). Anisotropy of the motion is evident. The fruit flies move more actively in horizontal than in vertical. The PDFs of z-scores of the horizontal velocity *z*_*vy*_ = (*vy*−*μ*_*vy*_)/*σ*_*vy*_ and of the vertical velocity *z*_*vz*_ = (*vz*−*μ*_*vz*_)/*σ*_*vz*_ have similar Gaussian shapes (but not Gaussian, see the upper panel of Fig 6b and 6c). Even though these PDFs deviate from Gaussian values at the tails, they suggest the mean velocity *μ*_*vy*_ and *μ*_*vz*_ of all configurations over time are approximately zero (see the lower panel of Fig 6b and 6c). That is, fruit flies in the arena do not show an overall polarisation, as shown in the inset of Fig 6a. Here the polarisation is defined as $\Phi =|\sum_{i=1}^{N}{\vec{v}}_{i}/v_{i}|/N$, where *N* is the number of fruit flies and ${\vec{v}}_{i}$ is the velocity of fruit fly *i*. The polarisation measures the degree of alignment of the directions of motion, where its value Φ ∈ [0, 1]. The polarisation of each configuration is small through time.

Fig 6 shows the statistics of velocity of fruit flies in the confined arena. These results indicate that the fruit flies’ kinematics are statistically similar with that of midges [23]. Midges are known to form swarms under visual markers, such as stagnant water [23, 24]. We here, however, observed that fruit flies in the arena flew almost randomly in the arena.

### 3.4 Spatial structure

**Fig 7:**
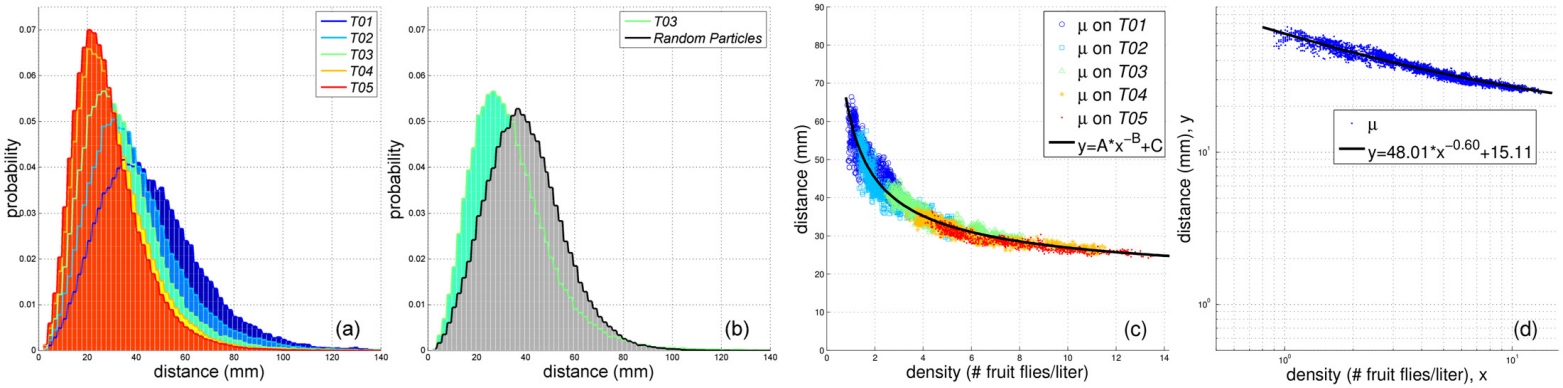
The NND distributions. The PDFs are represented by normalized histograms. (a) The PDFs ofNND distributions of all configurations. (b) The comparison between the PDFs of NND distributions of *T03* and the correspondent simulation (random particles). (c) The relationship between the average NND and the population densities at each moment of all configurations. (d) The average NND *y* as a power function of the population density *x*.

Even when hundreds of fruit flies were free-flying in the arena at the same time, no collision was ever observed. We speculate that the spatial organization of fruit flies may manifest the social structures. The clearest characterization of the spatial structure of fruit flies within a swarm is the probability distribution of the nearest-neighbour distance. The nearest-neighbour distance is the minimum Euclidean distance between a fruit fly and other fruit flies in a swarm. In the later text, we refer to the nearest-neighbour distance as **NND**. Table 1 shows the number of NNDs of each configuration. At each moment, there were many fruit flies landing on walls of the arena. Therefore, in order to avoid the bias introduced by those landing fruit flies (*i.e*. edge effects), we ignored a fly’s motion data if its distance to any walls of the arena is less than 20 mm. Fig 7a shows the PDFs of NND distributions of all configurations. It shows that the average NND and the deviation decrease as the population size increases. Moreover, considering the population size of *T05* is larger than that of *T04*, the similar PDFs of *T04* and *T05* indicate there exists an asymptotic saturation against the increasing population size.

In order to confirm whether there exists some biological forces or social forces among fruit fly swarms, we created a simulation system. The system simulated physical random particles (*a.k.a* Brownian motion in which social forces are absent) moving in a confined volume. The virtual volume was equal to the volume of the flight arena. The number of particles was equal to the number of fruit flies at each moment of each configuration. At the initial step, random particles were uniformly distributed in the volume. Each simulation ran on 3000 steps. We also evaluated the NND distribution of each simulation. Fig 7b shows the comparison between the PDF of NND distribution of *T03* and that of the correspondent simulation. The NNDs should follow a Poisson distribution for random particles. But the Poisson distribution fitting the distribution of the simulation compares poorly with that of fruit flies. The distribution of fruit flies obviously skews to the left. The difference between it and that of random particles is significant (*P* < 0.005, *t*-test).

Fig 7c shows that the NND decreases as the population density increases. The first dimension of each data element reported in Fig 7c is the average population density at a certain moment within a certain configuration, and the second dimension of each data element is the average NND at the moment within the configuration. It is obvious that the NND shows a power-law distribution against the population density. To quantify the relationship, we fit the data in Fig 7c with a decaying power function in the form

$$\begin{array}{c} y=A*x^{-B}+C \end{array}$$ (15)

where *x* denotes the population density and *y* denotes the NND. The constant *A* determines the overall scale of the variation with *x*, whereas *B* is the decaying rate constant. The constant *C* gives the asymptotic value for the average NND for all configurations. The interesting thing is the constant *C* denotes the exclusive distance between a fruit fly and its neighbours. Fig 7d shows the best fitting result. The best fitting *C* is *C* = 15.11 ± 0.89. It means that fruit flies have a high tendency to take the repulsive response when the distance between them approached *C* = 15.11 ± 0.89 mm. Considering the average body length of the fruit flies ($\tilde{l}=2.73$ mm), *C* can also be expressed as $C∼5\tilde{l}-6\tilde{l}$.

### 3.5 Acceleration towards nearest-neighbour

**Fig 8:**
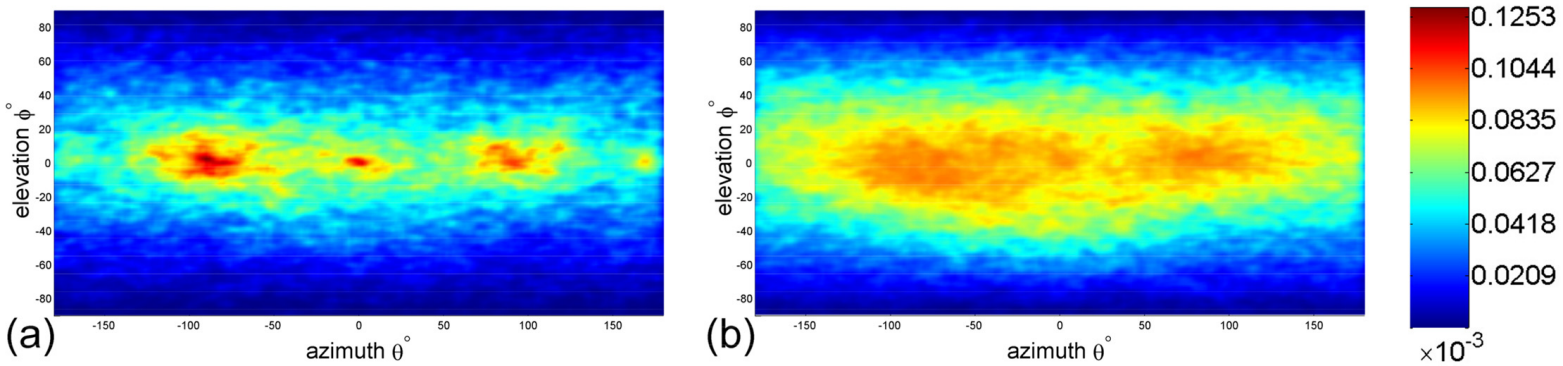
Probability density of the angular direction of fruit flies’ acceleration toward their nearest neighbours. For each fruit fly *i*, we defined the unit vector ${\vec{n}}_{i}$ in the direction of its nearest neighbour. We then computed the angular direction of the fly *i*’s acceleration with respect to the direction of its nearest-neighbour, ${\vec{n}}_{i}$. That is, we measured the azimuth and elevation of the fly *i*’s acceleration against the coordinate system which is defined by rotating the x-axis of the world’s coordinate system to ${\vec{n}}_{i}$. The azimuth is *θ* ∈ [−180°, 180°] and the elevation is *ϕ* ∈ [−90°, 90°]. The center point (*θ* = 0°, *ϕ* = 0°) corresponds to the direction of nearest neighbours $\vec{n}$. (a) All data were computed assuming the NND was less than $6\tilde{l}$. (b) All data were computed assuming the NND was greater than $8\tilde{l}$.

For each fruit fly in a swarm, $C∼5\tilde{l}-6\tilde{l}$ denotes the exclusive distance to other flies. If this result is significant, its effect should also be apparent in the motion statistics. We therefore measured the probability distribution of the angular direction of a fruit fly’s acceleration towards its nearest neighbours. Given a reference fruit fly, we computed the angular direction of its acceleration with respect to the direction of its nearest neighbour, $\vec{n}$ (see Fig 8, the legend). The angular direction is defined by two angles (the azimuth and the elevation). We repeated this computation by taking each fruit fly within a configuration as reference individuals, and mapped the probability density conditioned on the NND. In Fig 8a and 8b the NND condition (between $6\tilde{l}$ and $8\tilde{l}$) is omitted for clarity.

Fig 8a shows the distribution in which all data were computed conditioned on the NND is $<6\tilde{l}$; in this way, that of Fig 8b were computed conditioned on the NND is $>8\tilde{l}$. Both distributions are anisotropic, but the former has three obvious clusters. The left and right clusters (see Fig 8a) indicate a strong tendency towards a repulsive response. On the other hand, Fig 8b does not exhibit special manoeuvres. Fruit flies’ acceleration scattered when they met their nearest neighbours at distance $>8\tilde{l}$. Fig 8a shows another data cluster which is the cluster centering on ⟨0, 0⟩. It indicates that there were fruit flies accelerated directly towards their nearest neighbours. This manoeuvre can be thought as representing “chasing” behaviour.

## Discussion

In this paper, we detailed our tracking system which was designed for acquiring the motion data of individuals of fruit fly swarms through time. The fruit fly swarms were housed in a cubic flight arena. Three synchronized and calibrated high-speed cameras satisfy the minimum requirement of the system for resolving the ambiguity between targets. More cameras may increase the ability of resolving targets in a more dense population. The proposed tracking algorithm is easily tunable for more cameras because of the well designed measurement association algorithm. Beside, with the help of the “one-to-one” strategy, the proposed tracking algorithm is uncoupled from the population size. The computing time cost is linear to the population size. That is, the proposed tracking algorithm is suitable for tracking more targets depending on the capability of computers (multi-thread).

The probability distribution of the nearest-neighbour distances shows the spatial structure of fruit flies within a swarm. This distribution is significantly different to that of random particles (non social forces simulation). The average nearest-neighbor distance of all experimental configurations show the property of asymptotically approaching saturation. Further, by evaluating the distribution of angular direction of fruit flies’ acceleration towards their nearest-neighbours, we found the evidence proving the existence of the asymptotical distance. Fruit flies have a strong tendency to take the repulsive response when the distances between them approached $6\tilde{l}$ (the average body length is $\tilde{l}=2.73$ mm). This result is consistent with previous findings reported by Maimon *et al*. [10], who observed a strong decay in the probability of a fruit fly’s approaching small post if the post subtended a visual angle of ≈ 10° on the fly’s retina. At distance between fruit flies approaching $6\tilde{l}$, a fly subtends a visual angles of ≈ 10° on another fly’s retina. And therefore, fruit flies exhibit a high tendency to turn away from each others. Though there exists obvious interaction between a fruit fly and other flies, swarms of fruit flies in our experiments did not show an overall polarisation. On the average, unlike bird flocks [17], here the average polarisation is ≈ 0.02. It suggests that these fruit flies have little tendency to align their motion with their neighbours.

In conclusion, this study provided a detailed 3D tracking system for obtaining quantitative 3D motion data of individuals of the fruit fly swarms through time. The result of quantitative analysis shows that the fruit flies in a confined arena are not free particles. Behaviour rules exist in a fruit fly swarm and probably affect individual’s behaviours in the swarm. Understanding the detailed origin of their behaviours will be an interesting topic for future research.

## Supporting Information

S1 Fig The illustration of the equipment arrangement. (PDF)

S2 Fig The generative shape model of *Drosophila*. (PDF)

S3 Fig Summary of the raw data of typical configuration, *T03*. (PDF)

S1 File The performance evaluation of the proposed tracking method. (PDF)

S2 File The raw code. Readers can also download the release package using DOI: 10.5281/zenodo.13677
(ZIP)

S1 Video Demo video of the raw data of configuration *T01*. (MOV)

S2 Video Demo video of the raw data of configuration *T02*. (MOV)

S3 Video Demo video of the raw data of configuration *T03*. (MOV)

S4 Video Demo video of the raw data of configuration *T04*. (MOV)

S5 Video Demo video of the raw data of configuration *T05*. (MOV)
