# Supplementary material for: A Novel Method for Tracking Individuals of Fruit Fly Swarms Flying in a Laboratory Flight Arena
Source: PLoS One. 2015 Jun 17;10(6):e0129657. doi: 10.1371/journal.pone.0129657 (PMC4470659; doi:10.1371/journal.pone.0129657)
Supplement: S1 Fig — (PDF) [file pone.0129657.s001.pdf]

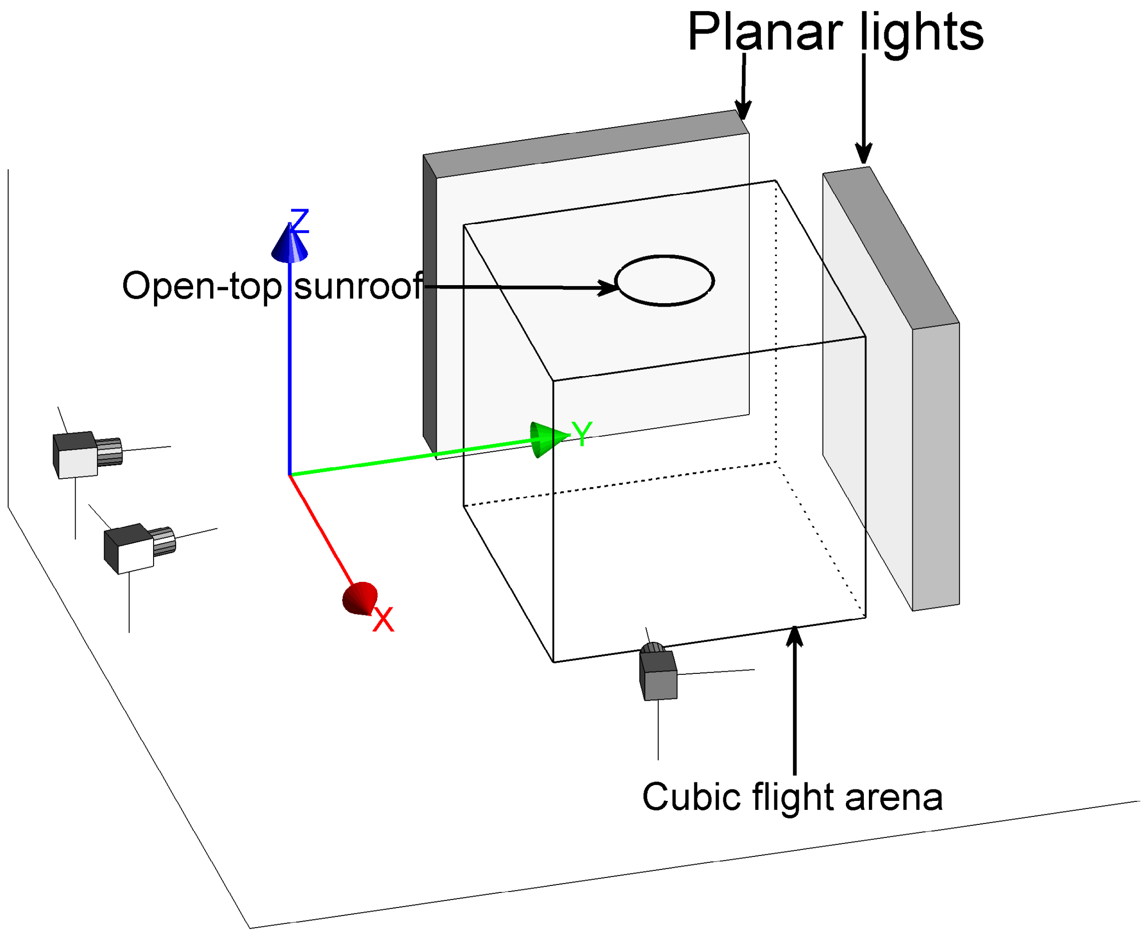

Figure 1. The illustration of the equipment arrangement. Two cameras were placed at one side of the arena. Another camera was placed at another side of the arena. These cameras were geometrically calibrated and hardware synchronized. The two planar lamps were orthogonally placed at the side of the arena opposite the cameras. Each lamp was made by LED arrays and covered by a diffusion sheet to generate gentle and flicker-free planar illumination. The world's coordinate system is color coded.
