# Supplementary material for: A Novel Method for Tracking Individuals of Fruit Fly Swarms Flying in a Laboratory Flight Arena
Source: PLoS One. 2015 Jun 17;10(6):e0129657. doi: 10.1371/journal.pone.0129657 (PMC4470659; doi:10.1371/journal.pone.0129657)
Supplement: S2 Fig — (PDF) [file pone.0129657.s002.pdf]

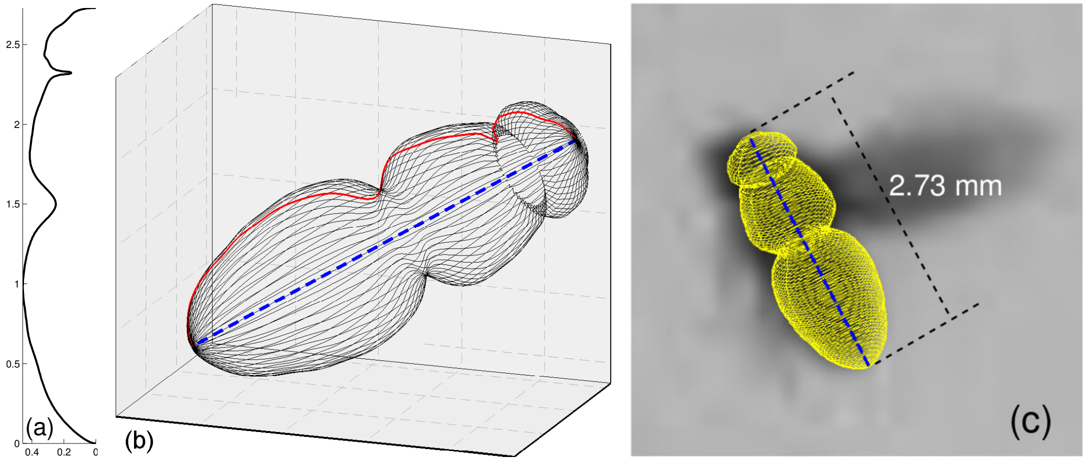

Figure 1. The generative shape model of *Drosophila*. (a) The profile curve, denotes by ρ. (b) The shape Υ^3 is generated by the profile curve ρ revolving around the *center axis* in 3D space. (c) An image of a fruit fly overlaid by the shape model. The average body length is $\tilde{l} = 2.73$ mm in our experiments.
