# Supplementary material for: A Novel Method for Tracking Individuals of Fruit Fly Swarms Flying in a Laboratory Flight Arena
Source: PLoS One. 2015 Jun 17;10(6):e0129657. doi: 10.1371/journal.pone.0129657 (PMC4470659; doi:10.1371/journal.pone.0129657)
Supplement: S3 Fig — (PDF) [file pone.0129657.s003.pdf]

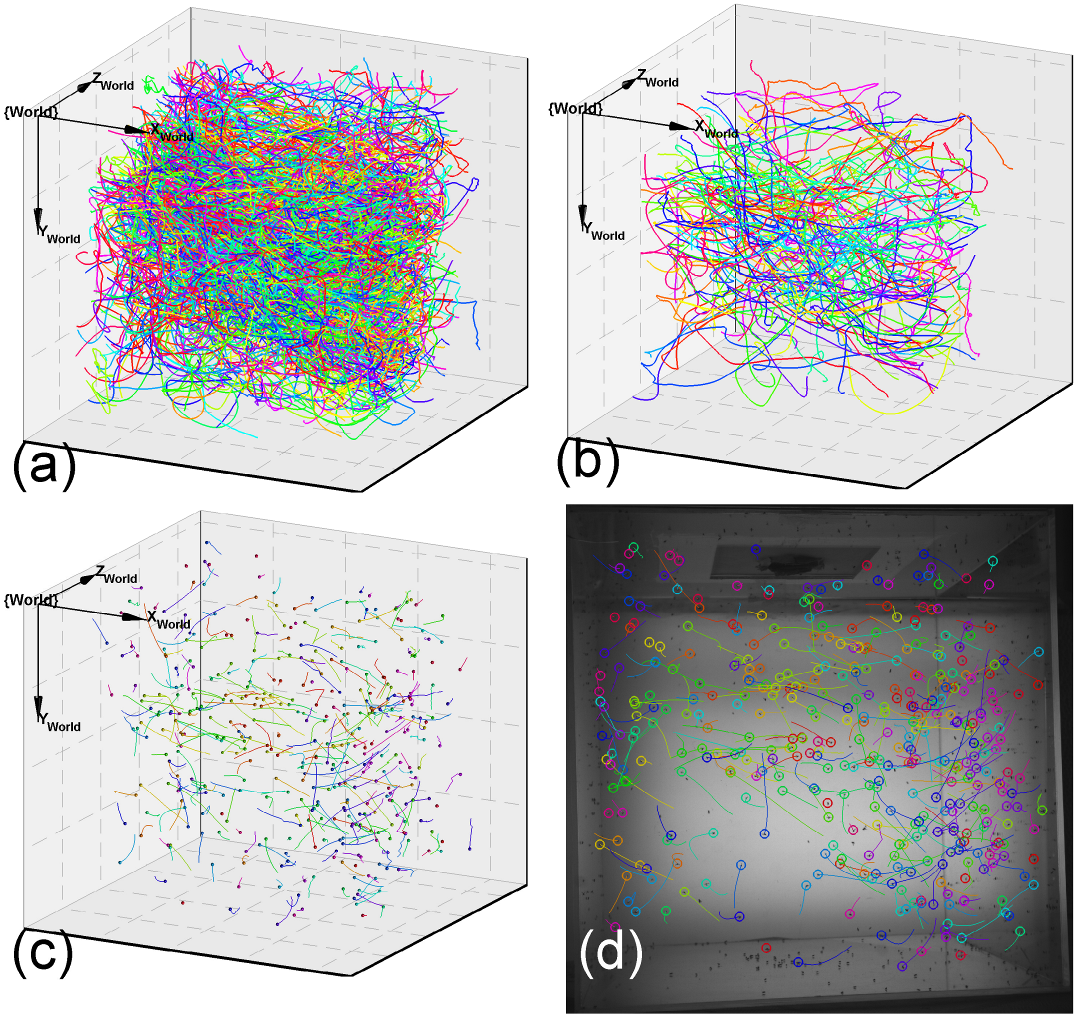

Figure 1. Summary of the raw data of a typical configuration, *T03*. Different trajectories are color coded. (a) All trajectories are longer than 1 second. (b) All trajectories are longer than 3 seconds. (c)-(d) Active targets at a certain moment with the 10 most recent locations are plotted in 3D space or projected onto 2D image, and different targets are color coded.
