# Supplementary material for: A Novel Method for Tracking Individuals of Fruit Fly Swarms Flying in a Laboratory Flight Arena
Source: PLoS One. 2015 Jun 17;10(6):e0129657. doi: 10.1371/journal.pone.0129657 (PMC4470659; doi:10.1371/journal.pone.0129657)
Supplement: S1 File — (PDF) [file pone.0129657.s004.pdf]

A Novel Method for Tracking Individuals of Fruit Fly Swarms Flying in a Laboratory Flight Arena

Xi En Cheng[1,3], Zhi-Ming Qian[1], Shuo Hong Wang[1], Nan Jiang[2], Aike Guo[2], Yan Qiu Chen[1,*]

1 School of Computer Science, Shanghai Key Laboratory of Intelligent Information Processing, Fudan University, Shanghai, China

2 State Key Laboratory of Neuroscience, Shanghai Institute of Biological Sciences, Chinese Academy of Sciences, Shanghai, China

3 Jingdezhen Ceramic Institute, Jingdezhen, China

∗ Corresponding author

E-mail: chenyq@fudan.edu.cn (YQC)

Tracking performance evaluation

We adopt the CLEAR MOT metrics [1] to evaluate the performance of location estimation on the dataset. The MOPT (stands for multiple object tracking precision) shows the ability of a tracker to estimate precise object positions.

$$\text{MOTP} = \frac{\sum_{i,t} d_t^i}{\sum_t c_t} \tag{1}$$

where d_t^i denotes the distance between locations which the estimated location and the ground truth are matched. Here c_t denotes the number of matching pairs at moment t. We choose $\hat{l} = 2.73$ mm as the matching criterion. It means two locations are matched if the distance between the estimated location and the ground truth is less than $\hat{l}$. The MOTA (stands for multiple object tracking accuracy) accounts for all object configuration errors made by a tracker, false positives, missed, mismatches, over all frames.

$$\text{MOTA} = 1 - \frac{\sum_t (m_t + fp_t + mme_t)}{\sum_t g_t} \tag{2}$$

where m_t, fp_t, and mme_t are the number of misses, of false positives, and of mismatches, respectively, at moment t. g_t is the number of targets at moment t.

Simulation data

The first step of simulation is to generate the ground truth. The arena for simulating is a cube of size $1e3 \times 1e3 \times 1e3$ units. Firstly, we generate the trajectory of each target using motion model. N objects are randomly distributed in the cube with velocities of random magnitude and random directions. The individuals interact via effective forces similar to molecular mechanics: a short-range inter-individual repulsion and a long-range attraction [2]. Secondly, given a target i, we compute its motion direction at each moment, $M(\theta, \phi)$. Thirdly, at each moment, the target's orientation $O(\theta, \phi)$ is computed as

$$O(\theta, \phi) = \begin{pmatrix} M(\theta) \\ \frac{\pi}{4} \end{pmatrix} + n, n \sim N(0, \Sigma) \tag{3}$$

where n denotes a small white noise. Targets' trajectories and their motion direction and their orientation form the ground truth. N range from 5 to 80 and the length of each trajectory is 1000 time units.

The second step of simulation is to simulate the multi-camera system and to generate image data.

Given a target's location and orientation, a shape which represents the target motion state can be generated (see S2 Fig.). At each moment, all targets represented by shapes are filmed by simulated cameras. Images filmed by these cameras through time form the dataset. Fig. 1 shows the arrangement of cameras and the snapshot of each camera. We employed the "Machine Vision Toolbox" [3] to simulate the three-camera system. The simulation data is provided with the raw code and can be downloaded from DOI: 10.5281/zenodo.13677

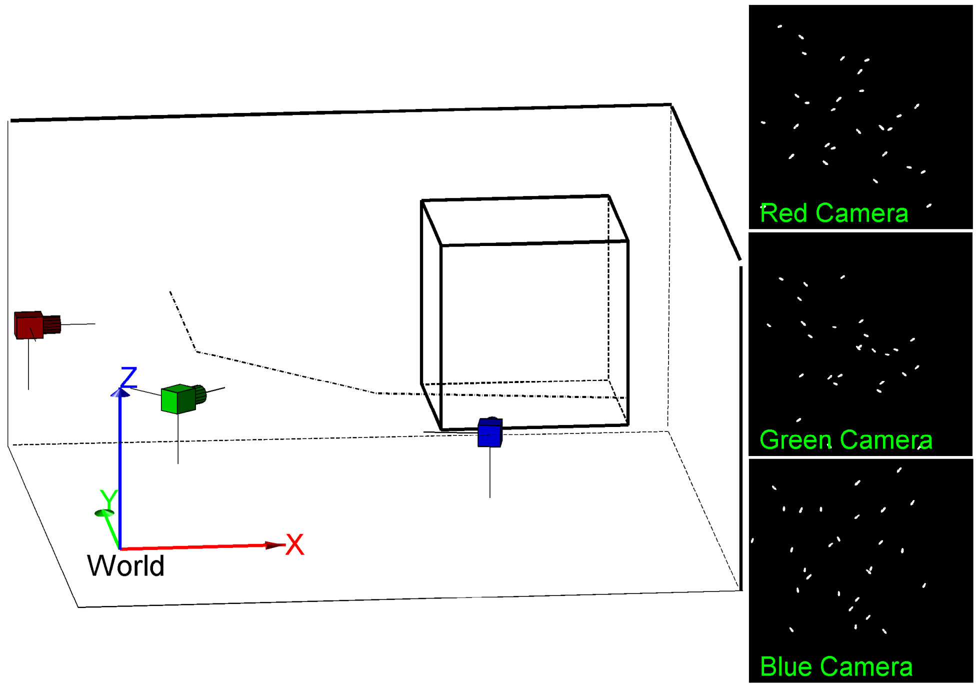

Figure 1. Cameras arrangement and snapshots. Three cameras were non-orthogonal placed.

Evaluation results

Each experiment is repeat three times, the reported results are the average score for each method. The proposed three-dimensional location and orientation tracking method, named "3D-LOT", validates a target's state at the end of a tracking iteration at each moment; it also rectifies the location and orientation of a target which is successfully validated. These validation and correction may eliminate error accumulation and propagation. Without orientation component, not only these validation and correction are disabled but also the particles' weight computation is degenerated, *i.e.* the orientation likelihood of particles' weight, p_{ol} (see the article, section 2.3.4), is disabled. The degenerated method

thereby estimates only 3D locations of targets and is named "3D-LT". Fig. 2 shows the results of performance evaluation of these two methods on the datasets. Fig. 2a shows the MOTP performance

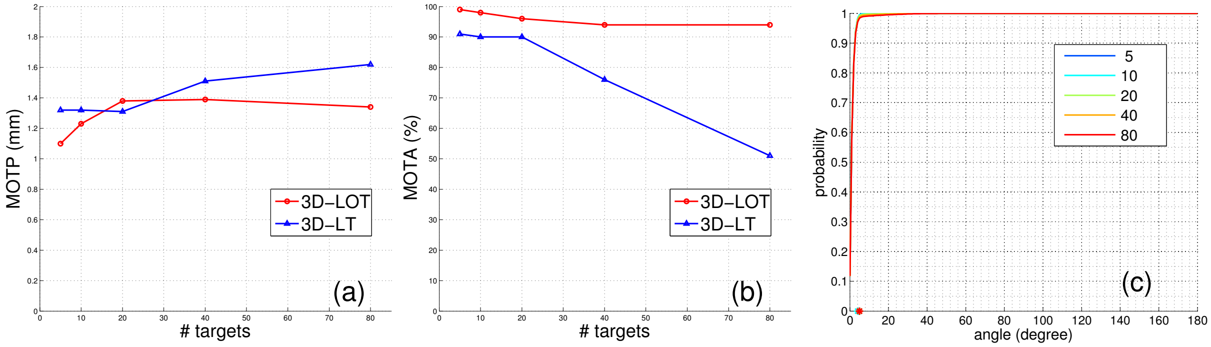

Figure 2. Performance evaluation. (a) The MOTP evaluation of 3D-LOT and 3D-LT on datasets. Smaller MOTP denotes better performance. (b) The MOTA evaluation of 3D-LOT and 3D-LT on datasets. Higher MOTA denotes better performance. (c) The performance of orientation computation of 3D-LOT on datasets. These are CDFs of angles distributions. Asterisks denote the 98% accuracy threshold (5° for all datasets).

of 3D-LOT and 3D-LT are not significantly different on datasets which have less objects (less than 40 objects). But as the population size increasing, *i.e.* the population density increasing, the 3D-LOT obtains better results significantly. On the other hand, Fig. 2b shows the MOTA performance of 3D-LOT is always better than that of 3D-LT. The validation process of 3D-LOT mostly contributes to the better MOTA performance, and the correction process contributes to the better MOTP performance of 3D-LOT, comparing to the 3D-LT. Of the particles' weight, the weight component of 3D-LOT, p_{ol}, increases the accuracy of the estimated result and thus promises the success of validation process. In a word, with the help of orientation component, the validation and correction processes prevent error accumulation and propagation.

Biological studies have reported many works on studying flight behaviours of fruit flies where a fly's orientation is a key source of information, such as [4–7]. Fig. 2c shows the performance of orientation computation of the proposed tracking method on datasets. Given a target i, we measured the accuracy of its computed orientation using the angle between the computed orientation and the ground truth throughout time. After all angles were computed, Fig. 2c shows the CDFs of angles distribution on datasets. As expected, the computed orientation is sufficiently accurate. Supposing we want to obtain 98% accuracy on targets' orientation, we only need to take a relatively small error interval, such as $[0°, 5°]$ for all datasets.

References

1. Bernardin K, Stiefelhagen R (2008) Evaluating multiple object tracking performance: The clear mot metrics. EURASIP J Image Video Processing . 2

2. Couzin ID, Krause J, James R, Ruxton GD, Franks NR (2002) Collective memory and spatial sorting in animal groups. J Theor Biol 218: 1-11. 2

3. Corke PI (2011) Robotics, Vision & Control: Fundamental Algorithms in Matlab. Berlin: Springer. 3

4. Card G, Dickinson M (2008) Performance trade-offs in the flight initiation of *Drosophila*. J Exp Biol 211: 341-353. 4

5. Ristroph L, Berman GJ, Bergou AJ, Wang ZJ, Cohen I (2009) Automated hull reconstruction motion tracking (hrmt) applied to sideways maneuvers of free-flying insects. J Exp Biol 212: 1324-1335. 4

6. Censi A, Straw AD, Sayaman RW, Murray RM, Dickinson MH (2013) Discriminating external and internal causes for heading changes in freely flying *Drosophila*. PLoS Comput Biol 9: e1002891. 4

7. Reiser MB, Dickinson MH (2013) Visual motion speed determines a behavioral switch from forward flight to expansion avoidance in drosophila. J Exp Biol 216: 719-732. 4
